# An Invitro Chewing Simulation Study Comparing the Wear Resistance Behavior of Polyetheretherketone-Layered Composite Crown and Ceramic-Layered Zirconia Crown

**DOI:** 10.7759/cureus.46439

**Published:** 2023-10-03

**Authors:** Divya Rupawat, Deepak Nallaswamy, Jayalakshmi Somasundaram, Dhanraj Ganapathy, Neeharika S, Saravanan Sekaran

**Affiliations:** 1 Prosthodontics, Saveetha Denal College and Hospitals, Chennai, IND; 2 Prosthodontics, Saveetha Dental College and Hospitals, Saveetha Institute of Medical and Technical Sciences, Saveetha University, Chennai, IND; 3 Prosthodontics, Saveetha Dental College and Hospitals, Chennai, IND; 4 Prosthodontics, Saveetha Institute of Medical and Technical Sciences, Saveetha University, Chennai, IND; 5 Prosthodontics, Saveetha Dental College and Hospitals, Saveetha Institute of Medical and Technical Sciences, Saveetha Univerity, Chennai, IND

**Keywords:** thermocycling, chewing simulator, wear resistance, health care, peek, zirconia

## Abstract

Objective: This study aimed to compare the wear resistance and color stability of fixed dental prostheses (FDPs) fabricated using two different materials: zirconia veneered with feldspathic porcelain and polyetheretherketone (PEEK) veneered with indirect composite. The assessment included samples subjected to thermocycling and wear simulation.

Methods: Two groups of FDPs were examined: one made of zirconia veneered with feldspathic porcelain (control and thermocycled) and the other made of PEEK veneered with indirect composite (worn and thermocycled). The samples were evaluated for wear resistance, antagonist wear, and color stability. Computer-aided design (CAD) software and a digital spectrophotometer were used for analysis.

Results: Zirconia veneered with porcelain demonstrated higher wear resistance compared to PEEK veneered with indirect composite. PEEK veneered with indirect composite exhibited significantly lower antagonist wear, indicating a protective effect on opposing teeth. There was no significant difference in color stability between the two groups, even after subjecting them to thermocycling and wear simulation.

Conclusion: The study concludes that FDPs fabricated with PEEK veneered with indirect composite may have lower wear resistance compared to zirconia veneered with porcelain. However, PEEK FDPs appear to be safer for antagonists due to reduced antagonist wear. Importantly, both materials exhibited similar color stability, making PEEK a viable alternative for FDPs when aesthetic appeal and antagonist protection are primary considerations.

## Introduction

For several decades, metal-ceramic fixed dental prosthesis (FDP) has been the gold standard in prosthetic and reconstructive dentistry with a positive evolution and development [1]. Survival of an FDP is considered successful when it remains free of all complications over the entire period of observation. According to a systematic review, the survival rate for metal-ceramic FDPs was estimated at 94.4% for over five years. This is due to their high standard mechanical properties and the clinically accepted quality of their internal and marginal adaptation [1]. A major challenge for the demand for aesthetics in FDPs still remains in certain clinical scenarios. Metal-ceramic FDPs tend to exhibit a greyish metallic hue and lack natural appearance due to the presence of opaque oxides [2].

With the evolution of all ceramic restorative materials and their enhanced optical properties, all-ceramic FDPs have become the new alternative to metal-ceramic FDPs when in need of aesthetics [3]. The main advantage of all ceramic materials is their ability to mimic the restoration similar to that of a natural tooth but with very limited indications such as the reconstruction of the anterior. Recent developments with the incorporation of alumina and zirconia have led to the progress of all-ceramic materials with favorable strength, biocompatibility, aesthetics, and a better clinical outcome with varied indications. Studies have proven that zirconia is one of the most commonly used substructure materials, which can be veneered with feldspathic porcelain successfully. Even though the restorations exhibited superior aesthetics, they are still prone to complications like fractures or chipping of the porcelain veneer and wear of opposing natural teeth due to their inability to withstand higher tensile stress and abrasive nature, respectively, which may cause failure of restoration on a long-term usage [3]

Polyetheretherketone (PEEK) has emerged as a high-performance polymer with a burgeoning presence in various medical dental applications, particularly in the realm of restorative dentistry. There are numerous compelling advantages that make PEEK an attractive choice for such purposes. PEEK has remarkable biocompatibility, rendering it well-suited for integration within the human body exhibiting a remarkable tolerance, avoiding allergic reactions or adverse tissue responses. This quality lends itself perfectly to applications like dental and medical implants, where compatibility with biological systems is paramount. In addition to its biocompatibility, PEEK offers exceptional mechanical properties. It exhibits high tensile strength and durability, making it a reliable option in restorative dentistry. With the ability to withstand the substantial forces generated during biting and chewing, PEEK-based restorations are synonymous with longevity. PEEK's lightweight nature is another advantage, particularly beneficial in dental prosthetics. Its reduced weight contributes to enhanced patient comfort, as it lessens the overall load of dental appliances and prostheses [4].

PEEK also possesses impressive chemical resistance. Resistant to the corrosive effects of chemicals and acids, it can endure the acidic environment of the oral cavity, thus minimizing the risk of degradation over time. Further bolstering its suitability for restorative purposes, PEEK exhibits minimal dimensional changes in response to temperature variations, ensuring that dental restorations maintain their shape and fit accurately. The ease of machining PEEK using standard dental laboratory equipment facilitates precise and customized restorations. Its versatility extends to various dental applications, encompassing crowns, bridges, removable dentures, and implant components, thereby offering a broad spectrum of treatment options. Another major advantage of using PEEK as a restorative material is its lower elastic modulus (4 GPa) closer to that of the alveolar bone, creating a positive cushioning effect while transferring occlusal load [5]. Beyond its mechanical attributes, PEEK has low thermal conductivity, contributing to reduced sensitivity to temperature changes and electrical conductivity. This quality minimizes the risk of galvanic reactions, ensuring patient comfort and safety [5].

PEEK can be seamlessly color-matched to natural teeth, enhancing the overall aesthetic appeal of dental restorations. Furthermore, PEEK's smooth surface resists the adherence of biofilms and bacteria, potentially reducing the risk of infection or inflammation around dental implants or restorations. This property contributes to both oral health and patient well-being. Finally, PEEK-based restorations tend to be less abrasive to opposing teeth compared to certain other materials, mitigating the risk of accelerated tooth wear [6].

Hence in a need to find alternative materials for FDPs and to overcome the complications of zirconia, the aim of our study is to compare and evaluate the wear resistance, abrasiveness, and color stability of multi-unit zirconia veneered with porcelain FDP versus a multi-unit PEEK veneered with indirect composite FPD using a chewing simulator, digital spectrophotometer, and universal testing machine, respectively, in an in vitro environment.

## Materials and methods

Study design****

The study included two groups with group 1 as zirconia coping with feldspathic porcelain and group 2 as PEEK coping layered indirect composite, which is subsequently divided into three subgroups, each with a control sample, thermocycled sample, and worn and thermocycled sample, respectively. The sample size was determined to be 18 per group to increase the level of significance using a G power calculation.

Sample preparation

An ideal tooth preparation with the specifications for an all-ceramic restoration was done on a mandibular second premolar and second molar on a Typodont model (Nissin, Japan). The first molar was removed to create pontic space. Following the tooth preparation, the model was replicated into multiple epoxy resin dies (Acculite Die Epoxy 8000 Fast Tan, American Dental Supply, Inc.). The three-unit epoxy resin die was then scanned in an intraoral scanner (Trios 3shape, Germany) and a three-unit FDP coping was digitally fabricated using the CAD software (3Shape, Germany). The standard tessellation language (STL) file was then milled in zirconia (CaroZiir S, UK) and PEEK (Bredent BioHpp, Germany) using five-axis computer numerical control (CNC) milling machines (IMES iCore 350i, Germany). The Zirconia (CaroZiir S, UK) was then sintered at 1450°C for 60 minutes. The Zirconia copings were layered with feldspathic porcelain (Ivoclar Emax, Ivoclar Vivaden, US), and PEEK crowns were layered with indirect composite (Bredent), which were then polished with polishing paste and rubber wheel according to the manufacturer's protocol [6,7]. A putty template was taken before the tooth preparations, and the same template was used to veneer porcelain and indirect composite over the samples to eliminate any bias toward the thickness of the veneer and maintenance of its natural anatomy. After layering, the crowns were stored at room temperature (25°C) for a minimum of 24 hours to check for any possible crack formations. The crowns were then sandblasted on the internal surface with aluminum oxide particles of 100 μm at 1 bar pressure for 10 seconds followed by bonding (Scotch Bond Universal, 3M Germany) and luting (Rely X 200 Universal Adhesive resin cement 3M, Germany) onto the epoxy resin.

Wear resistance using a chewing simulator

**Figure 1:**
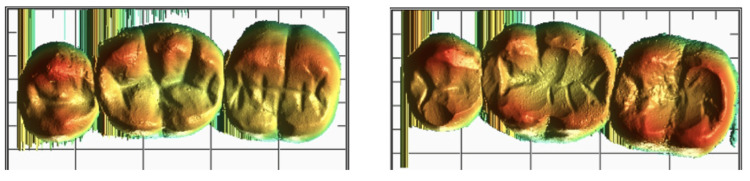
Pre- and post-wear of zirconia layered with porcelain FPD FPD, fixed dental prosthesis

**Figure 2:**
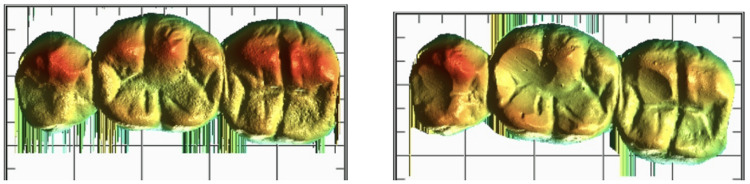
Pre- and post-wear of PEEK layered with indirect composite FPD PEEK, polyetheretherketone; FPD, fixed dental prosthesis

**Figure 3:**
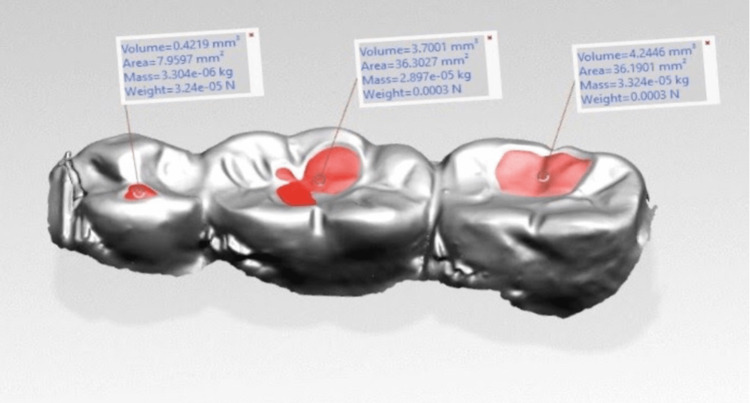
Superimposition of pre- and post-wear of samples in geomagicsTM software

Chewing simulators were used to evaluate the wear resistance, fatigue testing, and aging of the dental material over a prolonged period of time corresponding to more than 1 million cycles at a temperature range of -10 to 60°C. The chewing simulator (CS-4, SD Mechatronik, Germany) has four testing chambers within a thermocycling chamber. It has two moving parts, the vertical bar (Z-axis), and the horizontal table (X-axis). The samples were mounted onto the table, which can move back and forth. Customized antagonists measuring 4 mm in diameter (Steatite, SD Mechatronik, Germany) were connected to the vertical bar, which moves up and down with a load of 5 kg applied to the samples simulating a masticatory load intraorally. Thermocycling was done at a temperature of -10 to 60°C. The dwell time was 30 seconds and, the drain time was 10 seconds. The completion of each cycle was determined by one cold and one hot cycle. The filling time of the chamber was kept at 12 seconds. The total cycles of thermocycling were set to correspond with five years of aging as 12,00,000 cycles. The linear movement was applied to the samples in a bucco-lingual motion. The axis of movement of the antagonist was 2 mm in diameter on the X-axis, Y-axis, and Z-axis. The circular movement had a diameter of 4 mm, intrusion depth of 2 mm, and a speed of 20 mm/s. The evaluation of the wear can be done using a profilometer (LaserScanner LAS-20, SD Mechatronik), which analyses the surface of the sample to detect cracks and fine movements with a vertical sensor resolution of 0.8 μm. The samples from both groups were pretested in the laser scanner using the software and the color graph was obtained for a base template. After the samples had undergone through chewing simulator and thermocycling, they were analyzed for the amount of wear present in each sample using the laser scanner. The sample was inserted onto the self-centering mount. The measurement field was defined by a built-in camera. The data was exported as a “_scan” file, which was then converted into an STL format for superimposition of the pre-test and post-test samples (Figure 1 and Figure 2) in geomagics^TM^ CAD software (Figure 3). 

**Figure 4:**
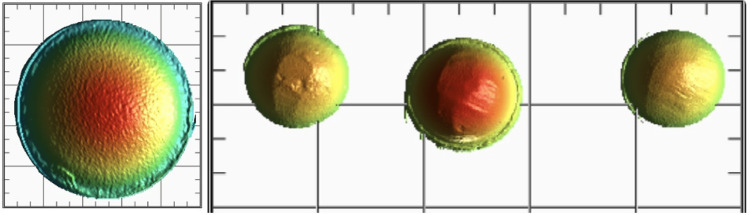
PEEK-indirect composite sample antagonist pre- and post-wear PEEK, polyetheretherketone

**Figure 5:**
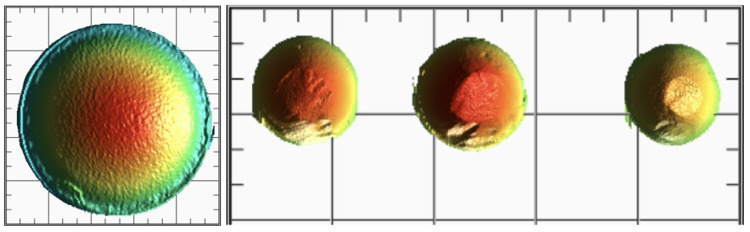
Zirconia-porcelain sample antagonist pre- and post-wear

**Figure 6:**
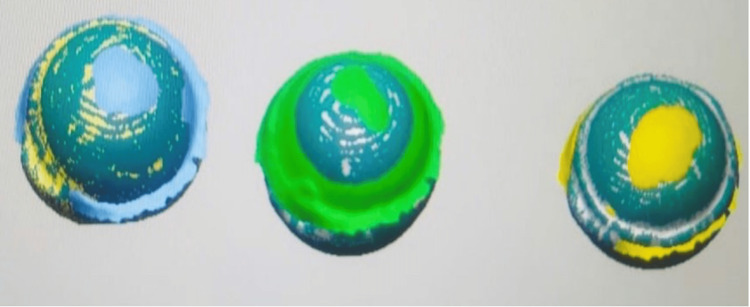
Superimposition of pre- and post-wear of antagonists in geomagicsTM software

Similarly, antagonists were also scanned before and after the samples were introduced to assess the antagonist wear (Figure 4 and Figure 5) followed by the superimposition of the samples in geomagics^TM^ software (Figure 6).

Color stability using a digital spectrophotometer

A spectrophotometer measures the intensity of light when it passes through a material. It captures the color and gives the data in terms of L*a*b* values which can also be derived as ΔE values for the color stability of the material. In* *our research, we utilized an ultraviolet and visible light digital spectrophotometer to assess the color stability of samples both before and after undergoing thermocycling. The color stability tests were conducted without exposing the materials to colored solutions like tea or coffee. The spectrophotometer was connected to a computer system for measuring color, while the software was employed to record spectrophotometric data and calculate the total color difference (ΔE) for each disc sample. Colorimetric measurements in the CIELab color space, capturing the L*, a*, and b* values, were taken against a white background. These measurements were conducted on each specimen, and the mean values for L*, a*, and b* were computed based on three separate measurements at different time intervals. All recordings of L*, a*, and b* values were conducted by a single examiner. To determine the overall color difference between the two instances in the three-dimensional L*a*b* color space, we applied Hunter's equation, ΔE = [(ΔL*)² + (Δa*)² + (Δb*)²]^(1/2), yielding a single value, ∆E.

Data analysis

The data was tabulated in Excel sheets. The variables antagonist wear (mm³​), material wear (mm³), and color stability (ΔE) were normally distributed. Thus, parametric tests, the independent samples t-test, were used to make group comparisons using the IBM SPSS software Version 20 (IBM Corp., Armonk, NY, USA).

## Results

The study compared the wear resistance of testing material and antagonists along with their color stability under a chewing simulator. 

Material wear

**Figure 7:**
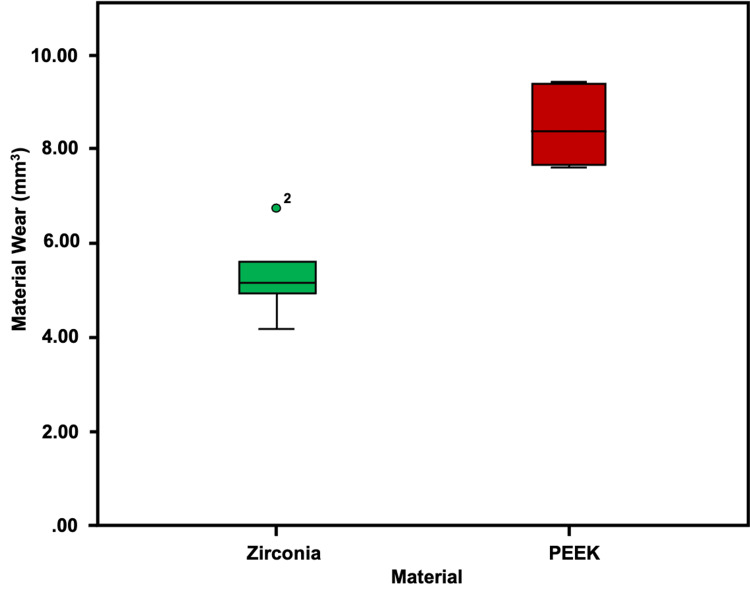
The box-and-whisker plot depicting the distribution of material wear (mm³) in the two groups. Zirconia-ceramic FDP post-wear is in green, and PEEK-indirect composite crowns post-wear is in red. PEEK, polyetheretherketone; FDPs, fixed dental prostheses

Zirconia layered with porcelain FDP was more wear-resistant than PEEK layered with indirect composite FDP under the influence of 12,00,000 cycles in the chewing simulator (p<0.001, independent samples t-test​) (Table 1, Figure 7).

**Table 1:** Wear resistance of the zirconia-ceramic and PEEK-indirect composite FDPs PEEK, polyetheretherketone; FDPs, fixed dental prostheses

Material wear in mm³​				
Group	Mean	Std. deviation	t-value	P-value
Zirconia-ceramic	5.288	0.848	0.881	0.004
PEEK-indirect composite	8.475	0.797		

Antagonist wear

**Figure 8:**
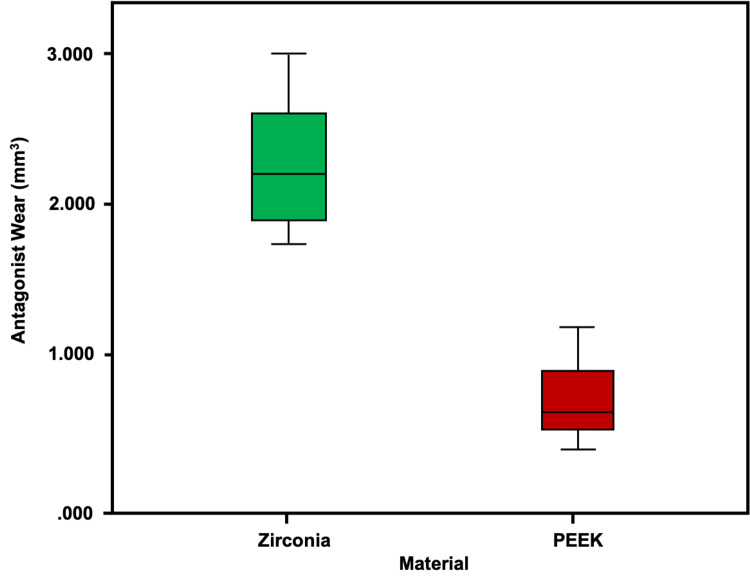
The box-and-whisker plot depicts the distribution of material wear (mm³) in the two groups. Zirconia-ceramic FDP post-wear is in green and PEEK-indirect composite crowns post-wear is in red. PEEK, polyetheretherketone; FPD, fixed dental prosthesis

The steatite antagonist wear was three times more with zirconia layered with porcelain FDP when compared to PEEK layered with indirect composite FDP with a significant difference (p<0.001, independent samples t-test​) (Table 2, Figure 8).

**Table 2:** Antagonist wear against zirconia-ceramic and PEEK-indirect composite FDPs PEEK, polyetheretherketone; FDPs, fixed dental prostheses

Antagonist wear in mm³​				
Group	Mean±SD	dF	t-value	P-value
Zirconia	2.285±0.459	0.320	6.941	0.000
PEEK	0.746±0.289			

Color stability

**Figure 9:**
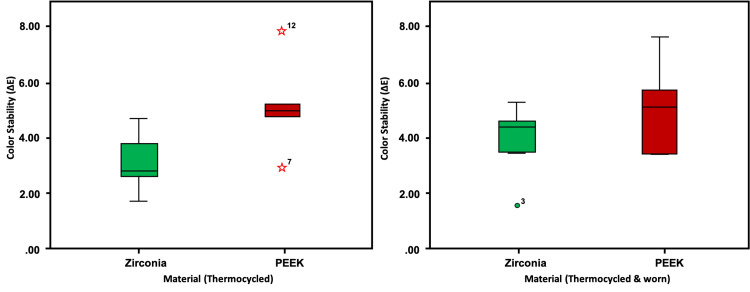
The box-and-whisker plot depicts the distribution of color stability over different timepoints. Zirconia-ceramic crowns in green and PEEK-indirect composite crowns in red

The ΔE values of color stability showed no significant difference when the zirconia layered with porcelain FDP and PEEK layered with indirect composite FDP underwent wear and aging in the chewing simulator (p>0.001, independent samples t-test​) (Table 3, Figure 9).

**Table 3:** Color stability of the samples

Color stability of the material (△E)				
	After thermocycling alone			
Group	Mean	Std. deviation	t-value	P-value
Zirconia	2.419	1.039	0.771	0.089
PEEK	5.086	1.583		
	After thermocycling and wear			
Group	Mean	Std deviation	t value	P-value
Zirconia	3.900	1.291	0.632	0.243
PEEK	5.016	1.616		

## Discussion

Properties of PEEK, zirconia, and composites have been investigated in many studies individually, but very few studies were found to test the performance of indirect composite layered over PEEK in comparison with zirconia [3]. Hence in this study, a PEEK layered with indirect composite FDPs was proposed to overcome some of the drawbacks associated with porcelain-veneered zirconia FDPs. Based on the results of our study, it is required to give special attention to the abrasive nature of any restorative material as it may damage the opposing antagonist collaterally. Wear is generally considered to occur due to occlusal interactions leading to complications like impaired chewing function, antagonist wear, and loss of durability of the prosthesis [8]. A randomized controlled trial (RCT) was conducted to check the survival rate of zirconia FDP and concluded that the survival rate of zirconia FDPs was 100% when compared with metal-ceramic FDPs [9]. A long-term study on feldspathic ceramic veneered zirconia restorations in the posterior tooth regions for over 10 years, indicates that there is evidence of wear over the period of time, which is in correlation with our study results supporting the wear of zirconia veneered with feldspathic porcelain samples [8]. Very few studies have been conducted on the wear resistance of the PEEK among which one study was conducted to verify the wear behavior among PEEK, nanohybrid composite, and PMMA-based material along with their antagonist wear [10]. The results concluded that specimens fabricated with PEEK have shown the lowest wear followed by composite and PMMA when tested laterally, with no significant wear on antagonists supporting the results of our study that PEEK veneered with an indirect composite was more protective toward opposing dentition and can be a better alternative for zirconia FDPs [10].

To evaluate the color stability of restorative material in simulation with an oral environment, a study was conducted on zirconia and PEEK copings veneered with an indirect composite under thermocycle and concluded that shades of the crowns have been affected by thermocycling, which is not in correlation with our study [7]. Various studies were conducted on the color stability of feldspathic porcelain and zirconia under different exposures of oral simulations and proved that these effects were higher on natural teeth and zirconia with feldspathic porcelain as least affected [11,12]. Since the recording of the color stability was made independent of the surrounding environment, this parameter had limited or almost no effect on the final color stability of the PEEK and zirconia crowns. It can be explained by the results of our study not being significant that the factor color stability will not be a complication toward the aesthetics in the future if a superior quality of veneering materials like indirect composites and feldspathic porcelains is used under substructures with enhanced optical properties (All ceramics and PEEK). Hence proving that PEEK layered with an indirect composite is as equally color stable as feldspathic porcelain over zirconia and further studies may increase the evidence to imply in clinical practice as an alternative to all ceramic restorations. 

Various confounding factors like the type of antagonist used, the quality of the material, the thermocycling process, the effect of the chewing simulator, and the bonding of veneering material may play an important role in concluding the results of the study. Also, to avoid any discrepancy in the uniformity of the samples especially while veneering the restorative materials, a preformed putty index was used on all samples to maintain equal thickness of the material. PEEK has a low surface energy, which may cause difficulty in bonding composites. With an improvement in adhesive properties of PEEK along with added mechanisms like airborne particle abrasion, plasma treatment, and chemical treatment, the bonding and bonding strength of composites have improved efficiently [3,6,13,14]. In our study, we have used airborne particle abrasion to improve the bonding of PEEK with indirect composite, which has proven to be an advantage while testing in an intraoral testing environment.

While testing in the chewing simulator every sample went through the same number of cycles (12,00,000 cycles, simulates five years) with a constant force of 200 Ncm​. The movement of antagonists and occlusion was established as a cusp to the fossa relation with the steatite balls similar to that of previous studies. Also to simulate the oral environment, the variable was maintained between -10 and 60°C in the thermocycling unit indicating that​ the chewing simulator was standardized according to the manufacturer for the present study. On the whole, this study was conducted following high-standard protocols and methodological sequences to provide positive and clinically applicable results. A long-term split mouth double-blinded randomized controlled clinical trial can be conducted to evaluate the wear pattern and color stability of the zirconia layered with ceramic and PEEK layered with indirect composite FDP.

Limitations

To standardize the study there is a need to develop a better chewing simulator that can simulate the anatomy and mandibular movements under normal oral conditions. In vitro trials are only useful to conduct preclinical trials of any newly introduced restorative material or designs. As enamel cannot be directly used as an antagonist due to errors in standardization, customized steatite balls were used. To correlate and support the relativity of values with a natural dentition, comparative evaluation can be done with the addition of a control group.

Statement of significance

This study was designed in search of new restorative materials as an alternative to all-ceramic restorations. Hence the study was conducted to compare the wear and color stability of a conventionally used zirconia layered with porcelain fixed prosthesis with a coping fabricated with PEEK and layered with indirect composite. To simulate the oral environment and undergo the aging process, the samples were tested in a chewing simulator with an in-built thermocycling unit. Results concluded that PEEK veneered with indirect composite FDPs showed less wear resistance than zirconia veneered with feldspathic porcelain FDPs. However, they were safer toward the opposing dentition by causing minimal wear of the antagonists. There is no significant difference in the color stability of PEEK layered with indirect composite FDPs and zirconia layered with ceramic FDPs indicating that either of the materials can be used in a long-term clinical scenario. With future advancements and further improvements in material properties, PEEK veneered with indirect composite restorations can show promising results as an alternative to all-ceramic restorations in clinical practice.

## Conclusions

With limitations, our study concludes that PEEK veneered with indirect composite FDPs showed less wear resistance than zirconia veneered with feldspathic porcelain FDPs. However, they were safer toward the opposing dentition by causing minimal wear of the antagonists. There is no significant difference in the color stability of PEEK layered with indirect composite FDPs and zirconia layered with ceramic FDPs indicating that either of the materials can be used in a long-term clinical scenario. With future advancements and further improvements in material properties, PEEK veneered with indirect composite restorations can show promising results as an alternative to all-ceramic restorations in clinical practice.
